# Bioengineering the human spinal cord

**DOI:** 10.3389/fcell.2022.942742

**Published:** 2022-08-26

**Authors:** Nisha R. Iyer, Randolph S. Ashton

**Affiliations:** ^1^ Department of Biomedical Engineering, Tufts University, Medford, MA, United States; ^2^ Wisconsin Institute for Discovery, University of Wisconsin—Madison, Madison, WI, United States; ^3^ Department of Biomedical Engineering, University of Wisconsin—Madison, Madison, WI, United States

**Keywords:** microfluics, micropattering, stem cell differentiation, genetic engineering, organoids, spinal cord, dorsoventral and rostrocaudal axes

## Abstract

Three dimensional, self-assembled organoids that recapitulate key developmental and organizational events during embryogenesis have proven transformative for the study of human central nervous system (CNS) development, evolution, and disease pathology. Brain organoids have predominated the field, but human pluripotent stem cell (hPSC)-derived models of the spinal cord are on the rise. This has required piecing together the complex interactions between rostrocaudal patterning, which specifies axial diversity, and dorsoventral patterning, which establishes locomotor and somatosensory phenotypes. Here, we review how recent insights into neurodevelopmental biology have driven advancements in spinal organoid research, generating experimental models that have the potential to deepen our understanding of neural circuit development, central pattern generation (CPG), and neurodegenerative disease along the body axis. In addition, we discuss the application of bioengineering strategies to drive spinal tissue morphogenesis *in vitro*, current limitations, and future perspectives on these emerging model systems.

## Introduction

**FIGURE 1:**
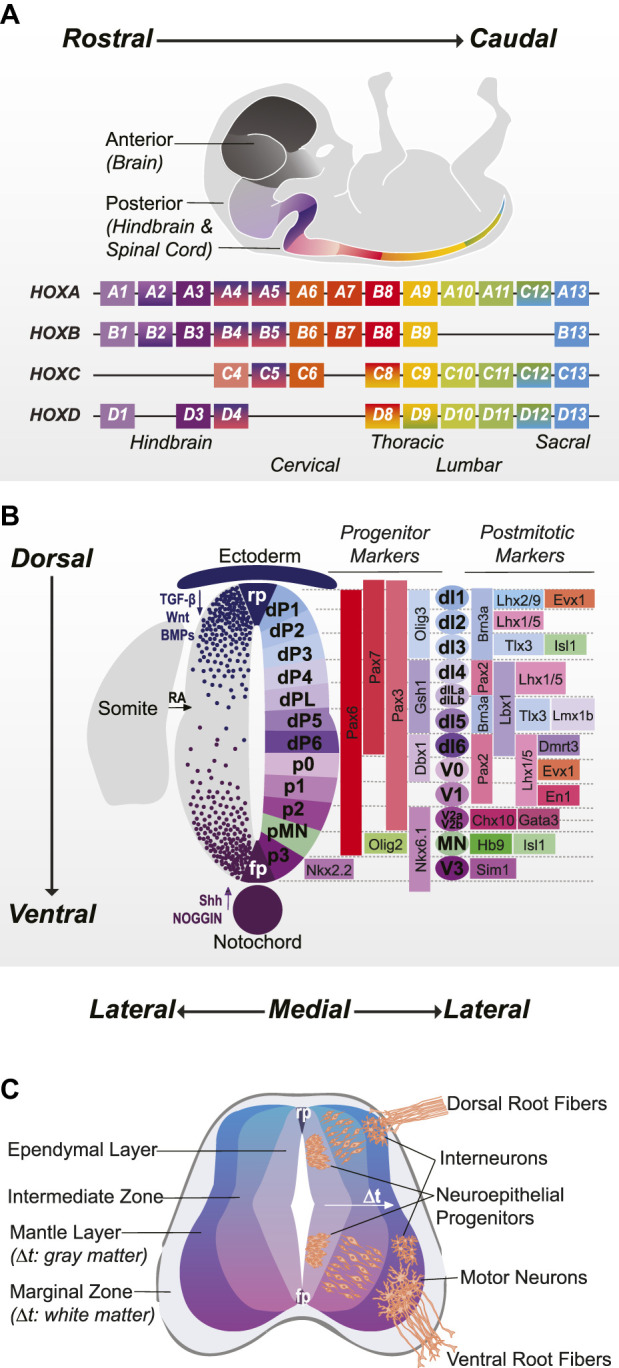
Three Axes of Spinal Cord Development: Rostrocaudal, Dorsoventral, and Mediolateral **(A)** Positional identity in the posterior CNS is defined by overlapping expression of HOX transcription factors: hindbrain (HOX1-4), cervical (HOX4-8), thoracic (HOX8-9), lumbar (HOX9-11) and sacral (HOX12-13). **(B)**. Dorsoventral patterning occurs in response to roof plate (RP) and ectoderm-derived TGFβ, BMP, and Wnt signaling and floor plate (FP) and notochord-derived Sonic Hedgehog (Shh) and Noggin signaling. Schematic shows 11 discrete progenitor domains (plus a lateborn dorsal progenitor domain) and corresponding post-mitotic cardinal neuron populations with characteristic transcription factor marker expression. **(C)** Once outside the ependymal layer, progenitors differentiate into post-mitotic neurons and migrate to their final settling positions in the mantle layer. The mechanisms that regulate birthdate are poorly understood but have significant influence on neuronal migration and projection patterns.

Since the first cerebral organoids were created in 2013 (Lancaster et al., 2013), organoids representing diverse CNS substructures have been optimized by manipulating signaling factors that mediate cell identity (Jacob et al., 2021). Replicating the spinal cord has posed a particular challenge. Spanning more than 25% of the length of the body, the spinal cord is a tubular structure consisting of 30 segments along the rostrocaudal axis. Each segment is comprised of anatomically distinct sensorimotor cell types defined along the dorsoventral and medio-lateral axes, which in turn form specialized circuits responsible for precise behavioral and sensory functions. Producing faithful spinal organoids thus requires integration of continuous, temporally defined orthogonal gradients of signaling factors that impart both positional and phenotypic information in three dimensions (Figure 1). In the past few years, facets of this task have been achieved in numerous studies, each reflecting different components of spinal cord biology. In this review, we focus on the two major axes of development—rostrocaudal and dorsoventral—as a framework to contextualize and evaluate these methodologies. We provide recent updates to our understanding of spinal cord neurodevelopmental biology, and how these findings influence emergent bioengineering strategies for generating human spinal organoids.

## Axial patterning in development and *in vitro*

Unlike the forebrain and midbrain which develop from the anterior epiblast, the hindbrain and spinal cord form as the embryo elongates from the caudal lateral epiblast. There, a bipotent population of axial stem cells called neuromesodermal progenitors (NMPs) differentiates into both the posterior neuroectoderm, which forms neural tube and neural crest derivatives, and the somites, which go on to form dermis, skeletal muscle, connective tissue, and vertebrae (Henrique et al., 2015). A balance between retinoic acid (RA), WNT and fibroblast growth factor (FGF) signaling contributes to the maintenance and proliferation of NMPs, driving axial extension and the acquisition of increasingly caudal *Hox* genes that confer positional identity (Figure 1A). Later, tail-bud secreted growth differentiation factor 11 (GDF11) serves to stimulate thoracolumbar and sacral *Hox* domains and repress rostral *Hox* genes (Liu, 2006). Neural plate specification is triggered by attenuation of WNT and increased RA signaling, prompting the differentiation from NMPs to region-specific neuroepithelial progeny (Gouti et al., 2014, 2017; Lippmann et al., 2015). Notochord-secreted NOGGIN, an antagonist of bone morphogenetic proteins (BMPs) and inhibitor of SMAD signaling further promotes neural induction and neurulation—the process by which the flat neural plate folds into the neural tube (Stottmann et al., 2006).

Commitment to an axial identity before neural induction is required for caudal spinal cord differentiation (Metzis et al., 2018). Thus, directed differentiation protocols for human pluripotent stem cells (hPSCs) that do not explicitly go through an NMP intermediate and instead apply neuralizing SMAD inhibitors before or in conjunction with caudalizing factors overwhelmingly result in hindbrain or rostral cervical identities (Amoroso et al., 2013; Du et al., 2015; Maury et al., 2015; Gupta et al., 2018; Bradley et al., 2019; Butts et al., 2019; Ho et al., 2021). Spinal cord organoids initiated with SMAD inhibitors are also rostrally oriented (Ogura et al., 2018; Andersen et al., 2020; Valiulahi et al., 2021; Lee et al., 2022). However, rostral HOX profiles in 2D and 3D can be shifted caudally by removing early SMAD inhibition and/or extending culture exposure to FGF and WNT signaling (Maury et al., 2015; Ogura et al., 2018; Duval et al., 2019; Andersen et al., 2020).

By decoupling the caudalization process from spinal cord patterning specifically, protocols for hPSC-derived NMPs constitute a replicable foundation for deriving diverse and discrete region-specific spinal tissues along the body axis. These protocols leverage FGF, WNT, and GDF11 signaling, which work synergistically to promote concentration and time-dependent activation of HOX genes *in vitro* (Gouti et al., 2014; Lippmann et al., 2015). Addition of RA and dual SMAD inhibition terminates HOX propagation, resulting in neural progeny expressing discrete HOX profiles (Gouti et al., 2014; Lippmann et al., 2015; Chasman and Roy, 2017; Verrier et al., 2018; Iyer et al., 2021; Mouilleau et al., 2021). Because NMPs are multi-lineage progenitors, organoids initiated from a discrete NMP pool can be patterned with hepatocyte growth factor (HGF) and insulin-like growth factor (IGF) to form spinal, mesodermal, and neural crest tissues together (Faustino Martins et al., 2020; Olmsted and Paluh, 2021, 2022). This is in contrast to cortico-motor “assembloids,” where region-specific organoids are fused after patterning (Andersen et al., 2020). Thus, though limited to a single axial location, NMP-derived trunk organoids enable modeling of human CNS–peripheral nervous system (PNS) co-development with their end-targets, including the primitive gut tube, skeletal muscle, and heart (Faustino Martins et al., 2020; Olmsted and Paluh, 2021, 2022). Despite a significant degree of variability between multi-lineage organoids derived using these methods, a major advantage is the ability to conduct long-term functional assessments in spinal and/or target tissue. Notably, Faustino Martins et al. and Andersen et al. measure calcium activity, muscle contractility, and network field potentials demonstrative of rudimentary CPG-like activity over 7–8 weeks in their neuromuscular organoids and cortico-motor assembloids, respectively. (Andersen et al., 2020; Faustino Martins et al., 2020).

“Gastruloids” that model the earliest stages of axial specification in post-implantation embryos are the best representatives of continuous rostrocaudal patterning in 3D (van den Brink and van Oudenaarden, 2021; Libby et al., 2021; Moris et al., 2020). Following a WNT-stimulated break in axial symmetry and the formation of NMPs adjacent to a tailbud-like signaling center, hPSC-derived gastruloids that begin as spherical aggregates elongate into tubular structures. Increasingly caudal, overlapping domains of HOX genes emerge with elongation, which become fixed as cells separate into distinct germ layers, including the neural tube, in response to endogenous signaling (Moris et al., 2020). While the comprehensive nature of gastruloids is impressive, their structural consistencies are variable and primitive. Particularly promising is the recent development of human “somitoids” that recapitulate robust somitogenesis in accordance with the segmentation clock (Sanaki-Matsumiya et al., 2022). By exposing NMPs to WNT signaling and low concentrations of Matrigel, Sanaki-Matsumiya et al. demonstrate reproducible elongation of somitoids with polarized structures containing only cell fates associated with the spinal column. Importantly, the level of WNT signaling instructed the ratio of somitic mesoderm (high Wnt) to neural tube (low Wnt) tissue (Sanaki-Matsumiya et al., 2022). Induction of a robust signaling center is key to the success of both gastruloids and somitoids, since localized protein patterning otherwise requires complex bioengineering strategies. As these methods evolve, inducing dorsoventral patterning, increasing neuronal differentiation efficiency and accelerating electrophysiological maturation are areas for significant improvement.

## Phenotype specification in development and *in vitro*

Locomotor and somatosensory spinal phenotypes are programmed along the dorsoventral axis of the neural tube. Ventral patterning begins in conjunction with neurulation, as Sonic Hedgehog (SHH) is secreted with NOGGIN by the notochord. These two factors also serve to suppress neural crest induction and dorsal patterning at early stages. As the neural tube closes, BMPs expressed from the roof plate promote dorsal patterning of the neural tube and proliferation and migration of neural crest cells from the neural fold. Dorsoventral patterning in the spinal cord is a classic model of morphogenetic activity, whereby molecular signals induce cellular responses dependent on the concentration of exposure; 11 discrete progenitor domains (5 ventral and 6 dorsal) emerge from the cross-repressive transcriptional interactions caused by the opposing SHH and BMP gradients (Figure 1B). Ventral progenitors (p0-p3 and progenitor motor neuron (pMN) domains) broadly give rise to neuronal populations responsible for locomotor coordination. The dorsal progenitors are split between BMP-dependent (dp1-dp3 domains) and BMP-independent (dp4-dp6) populations that correspond to proprioceptive and sensory neurons respectively. As progenitors divide laterally from the polarized apical surface of the neural tube, Notch-mediated suppression of neurogenesis fades, resulting in neurons with distinct birth dates (Götz and Huttner, 2005) (Figure 1C). Recent work has defined a shared transcription factor code for temporal emergence in the CNS, with gene expression analyses suggesting involvement of transforming growth factor beta 2 (TGFß2) signaling (Osseward et al., 2021; Sagner et al., 2021). Both HOX genes and neuronal birth date contribute to region-specific neuronal diversification and impact critical facets of spinal circuit organization including cell migratory patterns, projections, and synaptic targets (Dasen et al., 2005; Philippidou and Dasen, 2013; Marklund et al., 2014; Osseward et al., 2021; Rayon et al., 2021; Sagner et al., 2021). Thus, spinal phenotype is ultimately determined by “where” (rostrocaudal), “what” (dorsoventral), and “when” (mediolateral; temporal) cells emerge in development.

Numerous protocols in 2D and 3D have been developed for specific spinal populations by optimizing the concentration and duration of SHH and BMP signaling exposure. Most prevalent are high-efficiency protocols for MNs (Amoroso et al., 2013; Du et al., 2015; Maury et al., 2015; Ho et al., 2021), but there are increasingly methods available for ventral (Butts et al., 2019) and dorsal interneurons (Gupta et al., 2018) and glia (Bradley et al., 2019). Directed differentiation strategies that combine rostrocaudal and dorsoventral patterning are also on the rise (Lippmann et al., 2015; Iyer et al., 2021; Mouilleau et al., 2021). Because efficiency and speed of differentiation are prioritized in these protocols, spinal progenitors are often rapidly differentiated by adding DAPT, a Notch inhibitor, which precludes opportunities to generate and detect late-born neurons.

At the expense of differentiation efficiency, spinal cord organoids that exhibit dorsoventral organization have the advantage of the phenotypic diversity and laminar architecture present in the endogenous spinal cord. Patterning can be stimulated by exogenous signals in the media (Duval et al., 2019) or rely on the induction of roof plate or floor plate signaling centers (Ogura et al., 2018). In the case of the former, a concentration gradient of BMP4 or SHH is formed from the outside of the organoid inward, enabling stratified progenitor domains to develop (Duval et al., 2019; Andersen et al., 2020). Addition of DAPT rapidly converts these into to organized neuronal layers (Duval et al., 2019; Andersen et al., 2020). Alternatively, signaling centers can be induced in neural cyst-like organoids (Ogura et al., 2018). These exhibit an inverted polarized structure, whereby the apical layer containing progenitors is externally oriented, and neurons differentiate into the cell mass. With time, roof plate organizers stochastically form along the organoid edges and provide the signaling necessary for local dorsal domain development. Floor plate can be induced by adding sufficient SHH to the media, and the addition of exogenous BMP4 or SHH to the media can further dorsalize or ventralize the tissues (Ogura et al., 2018). Because the goal of these organoid models prioritize cell type derivation and organization, neuronal maturation and functional assessments are limited (Ogura et al., 2018; Duval et al., 2019).

While neither exogenous or endogenous signaling alone allows for co-development of the full spectrum of dorsal and ventral cell types, a combination seems sufficient. Andersen et al. demonstrate with scRNA-seq that spinal organoids patterned with exogenous SHH are capable of generating neurons from all spinal domains. Moreover, these spinal neurons form neural circuits receptive of cortical input and that synapse onto muscle to generate motor output (Andersen et al., 2020). As a result of relative size, the core of the organoids is likely shielded from SHH exposure and SMAD inhibition, enabling the spontaneous formation of roof-plate organizers. The resultant SHH-BMP4 cross gradients would thus allow simultaneous dorsal and ventral patterning (Andersen et al., 2020). In contrast, in the absence of either exogenous signaling or signaling center induction, spinal organoids default to intermediate phenotypes with sensory neuron-like electrophysiological characteristics (Lee et al., 2022). Whether any spinal organoids appropriately replicate temporal patterns of neuraonl emergence, akin to cortical layer stratification in brain organoids, has not been discovered. New marker availability in this regard should enable such determinations in the near future (Osseward et al., 2021; Sagner et al., 2021).

There remain multiple areas for phenotypic optimization. In addition to lacking organizational similarities to the native spinal cord, most spinal organoids comprise only of neural fates and attempt to model just the earliest stages of neurodevelopment. Extending culture duration, diversifying cell types and enhancing electrophysiology will be critical to further develop these platforms. Advancements in cortical organoid development serve as an example, where extended time in bioreactors and the addition of pertinent growth factors to promote astroglia (Trujillo et al., 2019; Velasco et al., 2019; Fair et al., 2020), microglia (Ormel et al., 2018; Bodnar et al., 2021), and oligodendrocyte fates (Madhavan et al., 2018; Marton et al., 2019) have been shown to improve functional maturation. Generating these tissues at scale for translation will also require standardization of methods across different hiPSC lines, adherence to good manufacturing practice (GMP) grade protocols and reagents, and thoughtful quality control methods and metrics.

## Bioengineering strategies to drive organization within spinal organoids

Current tissue engineering strategies for spinal cord organoids have comparable goals to those for other organ systems (Garreta et al., 2021; Hofer and Lutolf, 2021). These include reproducibility, development and maintenance of stereotypical 3D cytoarchitecture, and mimetic cellular composition and organization (Fu et al., 2021). As these initial objectives are met using single or combinatorial bioengineering approaches, the challenge will be to enhance the physiological maturation and lineage complexity within these tissues. Integration of descending brain stimuli, innervation of target tissues, and afferent sensory signals into multi-tissue spinal cord organoid platforms will eventually be necessary for holistic models that recapitulate the precise wiring and function of the human spinal cord *in vitro* (Giandomenico et al., 2019; Andersen et al., 2020).

### Spatial confinement using biomaterials

**FIGURE 2:**
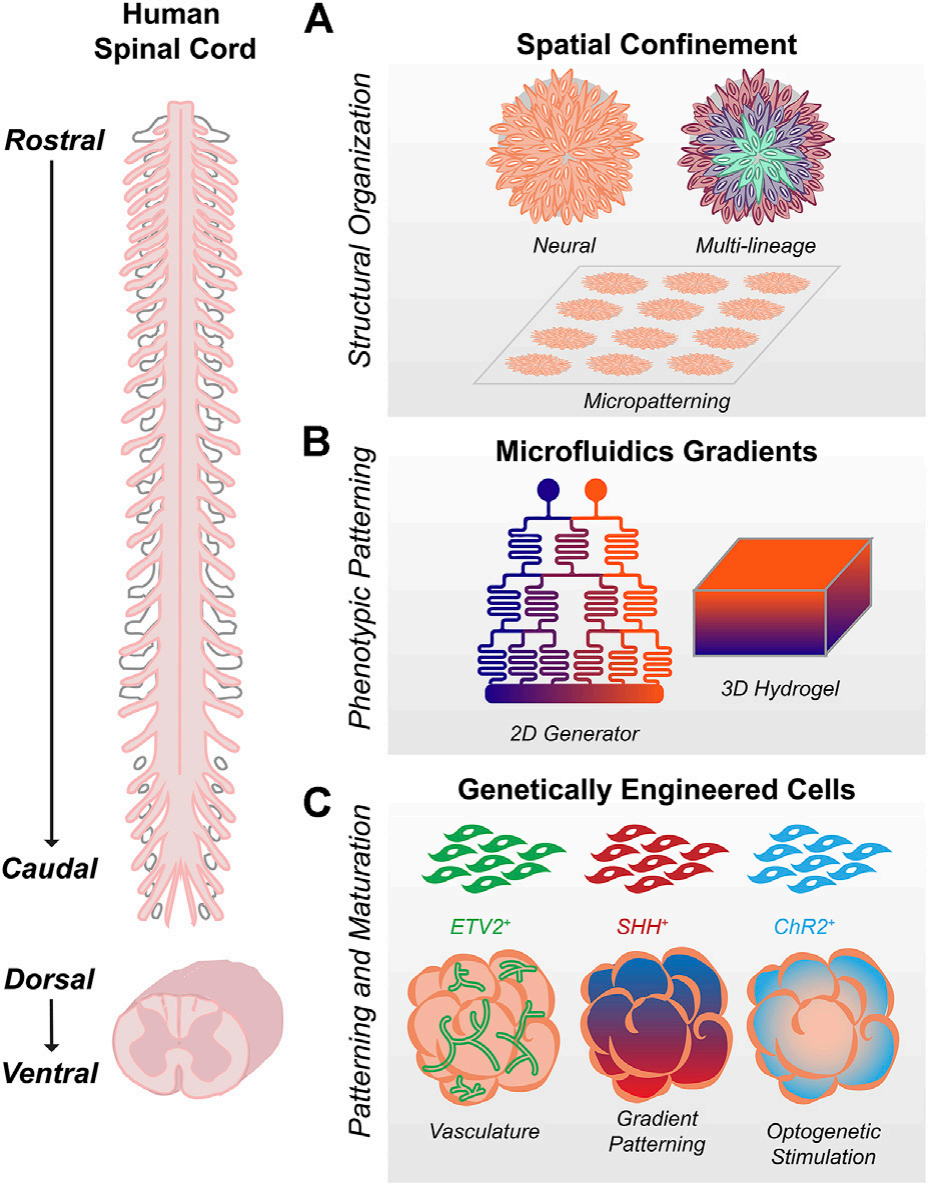
Bioengineering Strategies for Spinal Cord Organoids **(A)** Spatial confinement using biomaterials, including micropatterned substrates, enables control over tissue size and structure. Culture conditions can be used to refine whether organoids are wholly neural (including brain vs. spinal) or multi-lineage gastruloids representing multiple germ layers. **(B)**. Microfluidics in 2D or 3D can be used to generate orthogonal gradients capable of patterning the wide spectrum of cell types formed along the rostrocaudal and dorsoventral axes during spinal development **(C)** Addition of genetically engineered cells to organoids can enable vasculature, morphogen patterning, or optogenetic stimulation for improved cell type patterning and maturation.

2D micropatterning has emerged as a robust method to generate reproducible tissues with polarized cytoarchitecture (Figure 2A). In contrast to standard cell culture conditions, micropatterned surfaces enable control over tissue size and geometry, which significantly affect cell-cell signaling and the spatiotemporal emergence of different cell types. This confinement is sufficient to produce radial, self-organized germ layers mimicking the early embryonic events of gastrulation (Warmflash et al., 2014; Etoc et al., 2016; Chhabra et al., 2019; Minn et al., 2020). Directing micropatterned hPSCs to neural fates results in self-organized neuroectoderm specifically (Knight et al., 2015). Depending on culture conditions, layers representing neural plate, neural crest, placode, and surface ectoderm emerge (Knight et al., 2018; Xue et al., 2018; Britton et al., 2019; Haremaki et al., 2019; Sahni et al., 2021). However, most of these models represent anterior, not posterior neuroectoderm. Micropatterned substrates seeded with NMPs instead of hPSCs show optimal single-lumen polarization at smaller length scales, demonstrative of intrinsic biomechanical differences between anterior and posterior CNS tissues (Knight et al., 2018).

Micropatterned arrays in 2D can also be used as a template for 3D organoids. After attempting to caudalize hPSCs on micropatterned arrays, Seo et al. show that tissues detach to produce elongating spinal organoids that are organized along the dorsoventral axis (Seo et al., 2021). Mesodermal cells form a radial layer on the outer edge of the micropatterned colony, such that when detachment occurs, they remain localized to a single side of the organoid and act as a source for BMP signaling. Staining, single cell RNA-seq, and spatial RNA-seq data confirm that the resultant BMP4 gradient generates discrete dorsal through ventral progenitor domains except for the pMN and p3 domains, which likely require greater SHH than the organoids are able to produce endogenously (Seo et al., 2021). Kazbrun et al. demonstrate that stem cells reproducibly fold into an organized 3D structure with a single lumen when a layer of matrigel is added to their micropatterned substrates (Karzbrun et al., 2021). These tissues can subsequently be patterned with morphogens to create different structures including the amnion, neural tube, dorsal forebrain, and ventral floorplate (Karzbrun et al., 2021). The initial size of the micropatterned array determines neural tube shape, and as in 2D (Knight et al., 2018), folding and polarization can be interrupted by manipulating pathways associated with neural tube defects (Karzbrun et al., 2021). Though traditional suspension culture organoids are also capable of recapitulating neural tube morphogenesis (Lee et al., 2022), the reproducibility, ease of tissue processing, and clarity of imaging afforded by micropatterned substrates pave the way for translational high throughput neurotoxin and drug screening.

Geometric confinement in 3D using biomaterials is also gaining traction, as evidence in other organ systems point to the innate capacity of cells to self-organize and even undergo symmetry breaking when provided appropriate spatial and mechanical cues (Nelson et al., 2006; Zinner et al., 2020; Gjorevski et al., 2022). Sacrificial templates restricting differentiating neuroepithelial cells in a 3D mold have been explored as a means to generate cylindrical neural tube structures (McNulty et al., 2019). The 3D microenvironment can also influence dorsoventral patterning. Mechanoregulation of human forebrain organoids by stretch and interactions with matrix stiffness can enhance floor plate patterning (Abdel Fattah et al., 2021) and alterations in extracellular matrix parameters impact mouse stem cell-derived neuroepithelial cyst patterning (Meinhardt et al., 2014; Ranga et al., 2016). These are critical considerations as bioengineers design alternatives to Matrigel as an organoid culture substrate (Kozlowski et al., 2021).

### Microfluidics gradients

Complex morphogenetic gradients can be generated using microfluidics *in vitro*, enabling spatiotemporal control over rostrocaudal and dorsoventral organization in neural tissues (Figure 2B). By patterning WNT agonist on a microfluidic gradient generator in 2D, Rifes et al. generate human neural monolayers with continuous transitions between forebrain, midbrain, and hindbrain and establish a direct relationship between rostrocaudal organization and the steepness of the WNT activation gradient (Rifes et al., 2020). Similarly, to generate more caudal spinal cell types, Lim et al. use RA and GDF11 in a microhexagon gradient array to produce MNs from cervical through lumbar spinal cord (Lim et al., 2019). Demers et al. create four distinct gradients of RA, SHH, BMP, and FGF by designing microfluidic channels adjacent to the four sides of a cell-laden gel, modelling simultaneous rostrocaudal and dorsoventral patterning (Demers et al., 2016). Pairing microfluidics with 3D hydrogels can produce other morphogen gradients as well, including those for neural and non-neural interfaces (Cosson and Lutolf, 2014; Zheng et al., 2019; Li et al., 2021). Though throughput, length scales, and accessibility remain concerns, combining these types of gradient-generating platforms with geometric confinement methods in 2D and 3D offer ways to control both spatial and phenotypic organization (Marti-Figueroa and Ashton, 2017; Manfrin et al., 2019).

### Genetically engineered cells

In addition to their uses in lineage reporting and gene knockouts (Nie and Hashino, 2017), engineered cells have many potential applications for controlling organoid morphogenesis and maturation (Figure 2C). It is important to note that these strategies have been used primarily in brain organoids but may serve as a roadmap for future spinal cord work. hPSCs modified to express doxycycline-inducible SHH can be cultured as discrete signaling centers within larger spheroids (Cederquist et al., 2019). New CRISPR-Cas9 based technology enables photoactivation of transcription factors in 2D and 3D, allowing for optogenetic patterning of neural organoids with SHH (Nihongaki et al., 2017; Legnini et al., 2021). Compared to signaling centers induced spontaneously by exogenous organoid culture conditions, these represent intrinsic and spatially localized methods for morphogenetic patterning. Appropriate myelination, vascularization and electrophysiological maturation are also limited in the absence of relevant non-neural lineages. Mixing hPSCs engineered to ectopically express ETV2, Cakir et al. produce organoids with vascular-like networks that successfully integrate and are perfused by blood vessels *in vivo* (Cakir et al., 2019). Similar methods could be applied for astrocytes, oligodendrocytes, and microglia, given the prominent role they have in transcriptional and synaptic maturation (Porciúncula et al., 2021). Finally, optogenetics have been widely used to interrogate neural circuitry, but could stimulate *in vitro* electrophysiological maturation and the development of synchronous network activity (Shiri et al., 2019; Trujillo et al., 2019). By applying optogenetic stimulation to the human cortical organoid component of their cortico-motor assembloids, Andersen et al. induce both contractions and calcium spikes within the skeletal muscle component of the assembloid, demonstrating functional neural circuit formation (Andersen et al., 2020). The potential for engineered cells for interrogation or application of specific cell types is only limited by the availability of appropriate gene markers and biosensors.

## Applications and challenges for human health

The significant genetic and neuroanatomical differences between humans and animal models have hampered translational efforts for a variety of spinal cord conditions, including spina bifida (Juriloff, 2000), chronic pain (Burma et al., 2017), amyotrophic lateral sclerosis (Bonifacino et al., 2021), multiple sclerosis (Procaccini et al., 2015), and spinal cord injury (Nardone et al., 2017). However, most of the mechanisms underlying human spinal cord development and diversification remain unknown. Given that successful CNS regeneration in non-mammals relies on reactivation of key developmental signaling pathways (Cardozo et al., 2017), filling this knowledge gap is critical. Moreover, while the foundation of the neural tube is fully established within weeks of gestation, the spinal cord continues to mature past birth as corticospinal, motor, and sensory pathways develop and are dynamically refined. Spinal cord organoids represent an opportunity to study human-specific spinal cord circuits through both space and time as genome-editing, multi-omics, and live-imaging are applied to increasingly sophisticated models. Findings contribute not only to a basic understanding of human biology, but also strategies for pharmacological interventions, gene therapies, and cell transplantation. With patient-derived induced human pluripotent stem cells (hiPSCs), these organoids can reflect diverse genetic backgrounds and be used to investigate the molecular basis for patient-specific neurodegenerative phenotypes, advancing personalized medicine approaches. Finally, high-throughput microphysiological systems designed to screen for chemical/drug-induced neurotoxicity and novel pharmacological candidates have the potential to more effectively identify clinically relevant compounds while reducing the use of animals and high costs associated with pre-clinical and clinical trials. Though nascent, spinal cord organoids have shown remarkable progress in just a few short years and are expected to demonstrate even greater usefulness as tools for both basic science and translational research as these technologies continue to mature.

## Abbreviations

BMP, bone morphogenetic protein; CNS, central nervous system; CPG, central pattern generator; ECM, extracellular matrix; FGF, fibroblast growth factor; GDF11, growth differentiation factor 11; hepatocyte growth factor; hiPSCs, human induced pluripotent stem cell; hPSC, human pluripotent stem cell; IG, insulin-like growth factor; MN, motor neuron; NMP, neuromesodermal progenitor; PNS, peripheral nervous system; pMN, progenitor motor neuron; RA, retinoic acid; SHH, sonic hedgehog; TGFß2, transforming growth factor beta 2.
